# Impact of point-of-care clinical decision support on referrer behavior, imaging volume, patient radiation dose exposure, and sustainability

**DOI:** 10.1186/s13244-023-01567-7

**Published:** 2024-01-08

**Authors:** Amy L. Schranz, Dave T. Ryan, Raegan David, Graeme McNeill, Ronan P. Killeen

**Affiliations:** 1 Graduate Entry Medicine, University College Dublin, Dublin, Ireland; 2 Radiology Department, St. Vincent’s University Hospital, Elm Park, Dublin 4, D04T6F4 Ireland; 3 Radiology Department, Royal Victoria Eye and Ear Hospital, Dublin 2, Ireland; 4 School of Medicine, University College Dublin, Dublin, Ireland

**Keywords:** Radiology, Referral and consultation, Clinical decision support systems, Point-of-care systems

## Abstract

**Objectives:**

When referring patients to radiology, it is important that the most appropriate test is chosen to avoid inappropriate imaging that may lead to delayed diagnosis, unnecessary radiation dose, worse patient outcome, and poor patient experience. The current radiology appropriateness guidance standard at our institution is via access to a standalone web-based clinical decision support tool (CDST). A point-of-care (POC) CDST that incorporates guidance directly into the physician workflow was implemented within a subset of head and neck cancer specialist referrers. The purpose of this audit was to evaluate the imaging pathway, pre- and post-implementation to assess changes in referral behavior.

**Methods:**

CT and MRI neck data were collected retrospectively to examine the relationship between imaging referrals pre- and post-POC CDST implementation. Effective radiation dose and estimated carbon emissions were also compared.

**Results:**

There was an overall reduction in absolute advanced imaging volume by 8.2%, and a reduction in duplicate CT and MRI imaging by 61%, *p* < 0.0001. There was also a shift in ordering behavior in favor of MRI (OR [95% CI] = 1.50 [1.02–2.22], *p* = 0.049). These changes resulted in an effective radiation dose reduction of 0.27 mSv per patient, or 13 equivalent chest x-rays saved per patient, *p* < 0.0001. Additionally, the reduction in unnecessary duplicate imaging led to a 13.5% reduction in carbon emissions, *p* = 0.0002.

**Conclusions:**

Implementation of the POC CDST resulted in a significant impact on advanced imaging volume, saved effective dose, and reduction in carbon emissions.

**Critical relevance statement:**

The implementation of a point-of-care clinical decision support tool may reduce multimodality ordering and advanced imaging volume, manifesting in reduced effective dose per patient and reduced estimated carbon emissions. Widespread utilization of the point-of-care clinical decision support tool has the potential to reduce imaging wait times.

**Key points:**

• Implementation of the point-of-care clinical decision support tool reduced the number of patients who simultaneously had a CT and MRI ordered for the same clinical indication compared to a standalone web-based clinical decision support tool.

• The point-of-care clinical decision support tool reduced the absolute number of CT/MRI scans requested compared to the standalone web-based clinical decision support tool.

• Utilization of the point-of-care clinical decision support tool led to a significant reduction in the effective dose per patient compared to the standalone web-based clinical decision support tool.

**Graphical Abstract:**

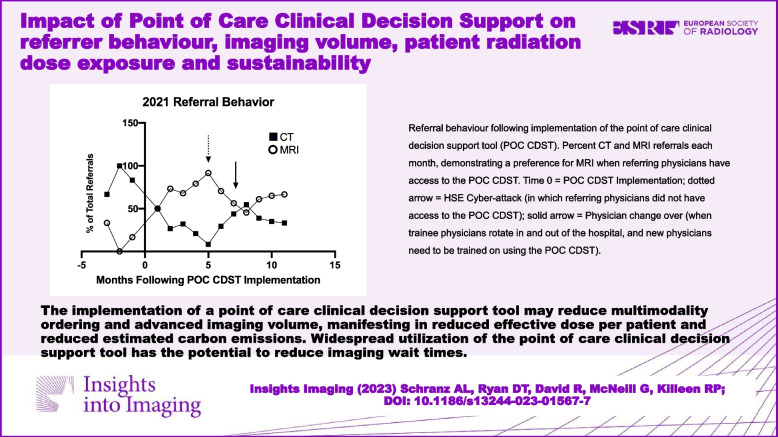

## Introduction

The use of medical imaging has exponentially increased over the last decade, and with it, an increase in inappropriate imaging referrals [1]. According to the Health Service Executive (HSE) in Ireland, approximately 2.5 million diagnostic imaging tests are performed in Ireland annually [2, 3], and in 2021, there were 226,166 people in Ireland waiting for imaging [4]. CT examinations in Ireland have almost doubled since 2009 and are associated with relatively high radiation doses [5]. For example, a study by Shao et al. found patients who developed thyroid cancer were more likely to have received CT scans, with 3% of cases associated with 1–3 scans, compared to 1.5% in controls [1]. Furthermore, a study by Tipnis et al. reported a link between thyroid cancer and the dose received from CT Neck scans [6], while Brenner et al. estimated that 1.5–2.0% of cancers in the USA might be attributable to CT scans [7]. In fact, it has been estimated that 20–50% of radiological examinations may be inappropriate or unnecessary [8]. When these procedures are unjustified, there is no net benefit to the patient. It is particularly concerning when patients receive unnecessary use of ionizing radiation when an alternative modality would have provided an accurate diagnosis [9]. Furthermore, inappropriate referrals lead to increased wait times for other patients, increased healthcare costs, and delayed diagnoses [10].

When referring patients to radiology, it is important that the most appropriate test is chosen initially, to avoid inappropriate or excess imaging. To prevent low-value, high-cost imaging, evidence-based referral guidelines are essential. The European Society of Radiology (ESR) developed the referral guidelines, *ESR iGuide*, based on the American College of Radiology (ACR) Appropriateness Criteria [11]. These guidelines use a rating scale, with 1 to 3 defined as “not usually appropriate”, 4 to 6 as “may or may not be appropriate” and 7 to 9 as “usually appropriate” [12]. With the use of iGuide, there may be improved referral appropriateness, reduction in unnecessary radiation exposure, and increased educational value [11]. Similarly, the iRefer guidelines, evidence-based imaging referral guidelines produced by the Royal College of Radiologists in the UK, have been adopted by the Irish Health Service Executive (HSE) [13]. This standalone web-based support tool has been available to referring physicians in Ireland since 2015 [14]. A retrospective review of radiograph referrals made through a single institution’s emergency department, in-patient, and general practitioners found 42% of referrals to be inappropriate prior to the implementation of web-based guidelines in Ireland. Following the implementation of the web-based support tool in Ireland, a similar number of referrals were found to be inappropriate (43%) [14]. Moreover, where paper referrals are still in use, referral data provided may be suboptimal and hinder the prevention of inappropriate referrals, that is, referrals that have missing, insufficient, or illegible information [15]. An incomplete referral does not allow the benefits of imaging to be balanced against the risks [16] and further adds to the radiology department workload [17]. Therefore, one strategy to prevent suboptimal and inappropriate referrals is the use of electronic referrals with clinical decision support implemented into the referring physician’s workflow [18].

A point-of-care (rather than standalone) clinical decision support tool (POC CDST) embedded into the referral workflow [19] was recently implemented for a subset of physicians at our institution. The purpose of this study was to evaluate the impact of the implementation of POC CDST and assess changes in referral behavior.

## Methods

### Data collection

This data assessment was approved by the institutional Audit Committee. The Radiology Information System (RIS) was used to compile anonymized data in Excel (Microsoft) pertaining to the referral process for head and neck (HN) imaging referrals from a separate specialist HN hospital. This included patient age, sex, presenting complaint, priority category, referring information (yes/no to department name, doctor name, and contact information on the referral), date of referral made, date imaging was ordered, date of exam, and date of final report. Specifically for referrals made through the POC CDST, the ESR iGuide score was recorded. Data was acquired for CT and MRI neck exams performed between January and December 2019 (Web-based CDST only) and between January and December 2021 (POC CDST and web-based CDST). All CT and MRI neck data were collected retrospectively, and all referrals came from the same Head and Neck Clinics in both cohorts. Both cohorts were age (*t*-test, ns) and sex (Fisher’s, ns) matched and were broadly disease-matched.

### Web-based versus point-of-care CDST

Referrals made through the web-based CDST pathway were paper referrals, where the referring physician may choose to access the tool (iRefer) through their institution’s website while completing the form. Referrals are then vetted and protocolled by the radiology team, at the origin site, before being faxed or emailed to the receiving institution’s radiology department for scheduling. Referrals made through the POC CDST pathway are made electronically through a cloud-based platform (xWave CDS, xWave Technologies 2021) that referrers can download onto their phones or access on the web. In the application, the referrer selects the imaging modality of choice followed by the clinical indication. The application then displays, where available, the ESR iGuide guidelines related to the specific inputted clinical indication, the appropriateness score, and the associated radiation dose exposure of the test selected. In addition to displaying the appropriateness score and relative radiation dose exposure of the test selected initially by the referrer, additional optional tests are displayed with their relative radiation dose and appropriateness scores. The referring physician may then continue with their initial choice of test or change to a different test based on the iGuide feedback the referrer is given directly within the application. Once the referral is submitted, the vetting radiology team is notified digitally. Vetting and protocolling are then performed by the radiology team within the application before the referral is available for scheduling. A digital chat function allowed any queries raised by either the radiology team or the referring team to be handled within the application.

### Point-of-care CDST training protocol

In conjunction with the Health Service Executive Digital Transformation team and sponsored by the Dean of the Faculty of Radiology in Ireland, a group of referrers were trained to use the platform to make radiology referrals. The user group was briefed on the use of evidence-based guidelines and appropriateness levels of imaging referrals. Initially, the radiographer services manager (RSM) within the radiology department was trained by the provider on how to use the application. On referrer sign-up, referrer training was provided by the RSM which took approximately 5 min. In addition, an on-demand tutorial was available within the application demonstrating the referral process (approximately 2 min in length). The time taken to make a referral through the application is typically under 1 min and access to customer support was also available via the provider (xWave Technologies).

### Suboptimal referrals

The number of patients imaged, including sex (%male; %female) and age (mean ± standard deviation) were collected. The expected referral volume for 2021 was calculated based on the number of scans per patient [20, 21] in 2019, which was 1.16. This was utilized to display the data in total counts. To assess the frequency of suboptimal referrals, the number of referrals with a legible clinical indication, complete referring physician information, and specific clinic return date were counted for both cohorts (2019 and 2021). The percent change in these categories was calculated as the difference between the expected and observed counts post-POC CDST implementation.

### Referrer behavior

To assess changes in referrer behavior, the volume of advanced imaging, duplicate imaging, and ratio of CT:MRI ordering were quantified. Duplicate imaging was defined as a patient receiving a CT and MRI within 3 months of each other for the same/similar clinical indication. The 3-month window was chosen due to the typical referral patterns in our clinical practice to allow for the clinical scenario where a referrer may request a CT neck (or MRI neck) and, based on its findings, later request an MRI neck (or CT neck) for further clarity. The percent change in these categories was calculated as the difference between the expected and observed counts post-POC CDST implementation. To investigate changes in radiation dose per patient, the effective dose was calculated as the number of CT neck exams performed multiplied by 1.76 mSv [22], divided by the total number of patients each year.

Estimated changes in carbon emissions were calculated by multiplying the number of CT and MRI exams performed by their estimated carbon dioxide equivalent (CO_2_e) per scan (CT = 9.2 kg/scan; MRI = 17.5 kg/scan) [23]. The number of saved trips to and from the hospital was estimated as the reduction in advanced imaging volume multiplied by 18 kg/trip [24] and was subtracted from the 2021 total.

### Statistical analysis

All statistical analyses were performed in GraphPad Prism, version 9.0 for Mac OS C (GraphPad Software, San Diego, CA, USA). A Fisher’s exact test was used to examine all parameters pre- and post-POC CDST implementation. Effective radiation dose and estimated carbon emissions between 2019 and 2021 were compared using an unpaired Student’s *t*-test. An alpha value of 0.05 was used for all statistical tests.

## Results

A total of 172 patients were referred for CT or MRI neck in 2019, resulting in 199 referrals. Following the implementation of the POC CDST in 2021, a total of 211 patients were referred for imaging, resulting in 224 scans. Details of patient demographics can be found in Table 1. The HN imaging referral pathway was predominantly for suspected HN cancers (HNC), with a small number of non-malignant referrals. There was a 23% increase in patient volume and a 13% increase in imaging referral volume in 2021.

**Table 1:** Patient and referral demographics

	**2019**	**2021**
**# Patients**	172	211
Sex %M(%F)	61 (39)	60 (40)
Age (mean ± SD)	56 ± 17	56 ± 17
**# Referrals**	199	224
%Web/%POC	100/0	59/41
**Clinical indication (%)**		
Known HNC	16	24
Suspected HNC/recurrence	4	7
Mass/swelling/lesion	63	38
Symptomatic	14	26
Other	3	5

*HNC* Head and neck cancer, *Symptomatic* Dysphagia/odynophagia, dysphonia/hoarseness, vocal cord palsy, foreign body/sensation/globus, otalgia, neck/throat pain, obstructive sleep apnoea, hearing loss, pulsatile tinnitus, malignant otitis externa, Horner’s syndrome, or salivary gland disease; *Other* Hyperparathyroidism, preoperative functional endoscopic sinus surgery, query nerve injury, trauma to the neck, suspected oesophageal perforation, suspected ocular ischemia, chronic laryngitis, exposed auditory canal, postop-scar enlargement, query Eagle syndrome, query trigeminal neuralgia, or sarcoidosis

### Suboptimal referrals

The number of illegible clinical indications (on paper referrals), incomplete referring physician information, and specific return date can be found in Table 2. Referring physician information was counted as incomplete if the referring department, physician name, or contact information was missing. Incomplete referrer information and specific return date significantly decreased following implementation of the POC CDST, *p* < 0.0001 and 0.002 respectively. There was a reduction in the expected observed rate of referrals with illegible clinical indications in 2021 by 50% but this was not significantly different, *p* > 0.05.

**Table 2:** Change in referral behavior: expected and observed

		2019	2021			
			Expected	Observed	% Reduction	*p*-value
Referrals (*n*)	Illegible indication	12	14	7	50	0.16
	Incomplete referrer information	165	202	112	44.5	< 0.0001*
	Missing return date	36	44	23	47.7	0.02*
Scans (n)	Total	199	244	224	8.2	< 0.0001*
	Duplicate Ordering	54	66	26	61	< 0.0001*
	CT	97	119	87	-	-
	MRI	102	125	137	-	-

Expected calculated using 23% increase in patient volume

* denotes significant *p*-value

### Referral behavior

**Fig. 1:**
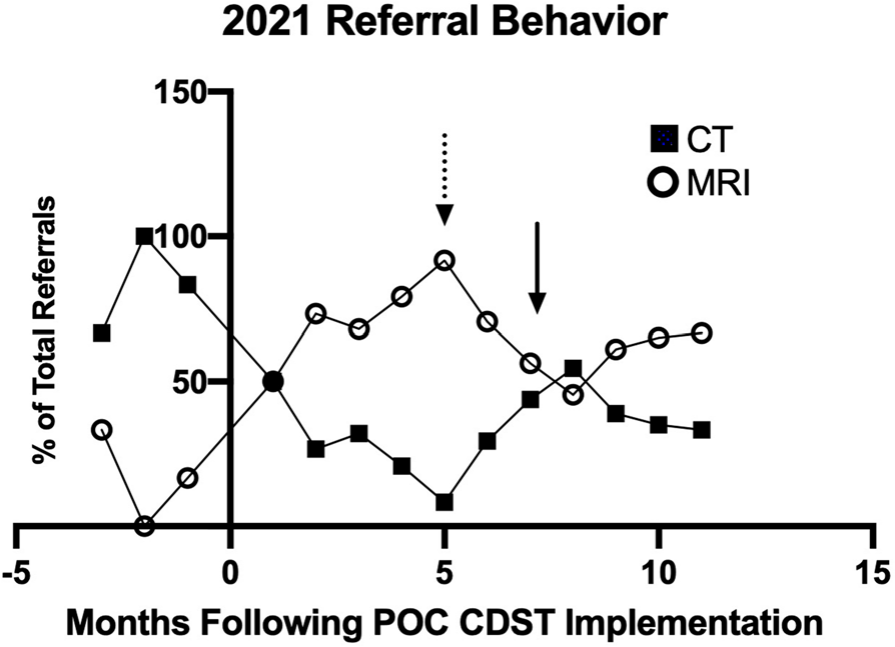
Referral behavior following implementation of the point-of-care clinical decision support tool (POC CDST). Percent CT and MRI referrals each month from the web-based and POC CDST, demonstrating a preference for MRI when referring physicians have access to the POC CDST. Time 0 = POC CDST Implementation; dotted arrow = HSE Cyber-attack (in which referring physicians did not have access to the POC CDST); solid arrow = physician changeover (when trainee physicians rotate in and out of the hospital, and new physicians need to be trained on using the POC CDST). The graph shows that following POC CDST implementation (Time 0), patients are preferentially referred for MRI. Following the HSE Cyber Attack in May 2021 (dotted arrow), there was a switch in referrer behavior in favor of CT in the absence of the POC CDST, when all new referrals were temporarily paper. Of note, the change in behavior, in favor of MRI, is again observed with the new referring physicians following physician changeover in July (solid arrow), as the POC CDST is reintroduced

The expected imaging volume in 2021 was calculated using the 23% increase in patient volume and can be found in Table 2. There was an overall reduction in absolute advanced imaging volume by 8.2% (*p* < 0.0001) and a reduction in unnecessary duplicate imaging by 61% (*p* < 0.0001). Patients who simultaneously had a CT and MRI ordered for the same clinical indication decreased by 73% (*p* = 0.006). There was also a shift in ordering behavior from 2019 (web-based CDST) to 2021 (POC CDST) in favor of MRI, odds ratio (OR) [95% CI] = 1.50 [1.02–2.22], *p* = 0.049. When comparing the web-based CDST (53% MRI; 47% CT) versus the POC CDST (73% MRI; 27% CT) in 2021, the OR [95% CI] was 2.4 [1.3–4.1], *p* = 0.005, of being referred for an MRI when POC CDST was used. The percentage of CT and MRI referrals made each month throughout 2021 is shown in Fig. 1. The HSE cyber-attack and physician changeover (when trainee physicians rotate in and out of the hospital) are plotted as well.

In 2019, the effective radiation dose was 0.99 mSv/patient, and following POC CDST implementation, the effective dose was significantly reduced by 0.27 mSv per patient (*p* < 0.0001) to 0.73 mSv/patient. Additionally, the reduction in unnecessary duplicate imaging led to 1.7 kg CO_2_e emissions saved per patient in 2021, or a 13.5% reduction in Carbon Emissions (*p* = 0.0002).

## Discussion

The purpose of this study was to evaluate the head and neck (HN) imaging pathway, pre- and post-POC CDST implementation to understand the pathway and assess changes in referral behavior. There was an overall switch in referral behavior following the implementation of the POC CDST within a subset of referrers, in favor of MRI over CT, and this reflected as a reduction in effective radiation dose per patient in the same patient population. There was also a significant reduction in imaging comparing the expected and observed number of orders, suggesting a reduced workload on the radiology department, as well as a reduction in overall estimated CO_2_e emissions. Using estimates from the literature [23, 24], approximate carbon emissions per patient were calculated pre- and post-POC CDST and we found a 13.5% reduction in estimated carbon emissions due to eliminating inappropriate duplicate imaging. This reduction is important because the government aims to reduce carbon emissions by at least 30% by 2030 [25]. Therefore, there is a need to prioritize reducing the carbon footprint of the healthcare sector, while maintaining patient care and ensuring patients receive the most appropriate imaging for their clinical problem. To help ensure the correct imaging modality is chosen, it is important that complete information is provided within the referral. Suboptimal referrals can lead to unjustified radiological examinations, the consequence of which may be subjecting the patient to the risks of the modality (e.g. ionizing radiation) without the benefit [15]. In this study, 83% of web-based CDST (paper) referrals had incomplete referrer information in 2019; that is the referral was missing either the referring department, name, or contact information. Contrast that with 50% of referrals following the implementation of the POC CDST in 2021. This increase in complete referrals is due to all referrals made through the POC CDST being complete. Furthermore, unjustified examinations can lead to further imaging which may ultimately delay diagnosis and treatment or result in patient mismanagement [15]. In this study, it was found that 14% of the imaging volume was due to patients being referred for both CT and MRI for the same or similar indications when referrers had access to only the web-based CDST. Following the POC CDST implementation, this imaging duplication was reduced by 61%. It was also observed that referrers were 2.4 times more likely to refer patients for an MRI neck when using the POC CDST versus the web-based CDST in 2021. Therefore, integrating the referral guidelines into the physician workflow reduced unnecessary imaging and caused a change in ordering behavior. This change is demonstrated in Fig. 1, where following POC CDST implementation, patients are preferentially referred for MRI. Interestingly, following the HSE Cyber Attack in May 2021, there was a switch to favoring CT, when all new referrals were temporarily paper and referring physicians did not have access to the POC CDST. Of note, the change in behavior, in favor of MRI, is again observed with the new referring physicians following physician changeover in July, as the POC CDST is reintroduced. Moreover, this change led to a 0.27 mSv/patient effective dose reduction, or 13 equivalent chest x-rays saved per patient [22]. The effect of the POC CDST on reduction in imaging volume, radiation exposure, and carbon emissions is likely underestimated given the interruption in its use related to the HSE cyberattack that occurred during the 2021 period under assessment.

While clinical evidence regarding the use of CDST for imaging appropriateness is heterogenous [26], POC CDST is postulated to overcome barriers to guideline implementations such as clinician knowledge, attitude, and behavior [27]. Studies have shown that one of the most effective ways to address these barriers is by providing the relevant guidelines during the clinician-patient interaction [28]. When clinicians were provided with decision support within their workflow, adherence to guidelines improved as compared to when a separate search was required to obtain guidelines (84% versus 37%) [27]. This is similar to the difference between a POC CDST (embedded in the workflow) and a web-based CDST (requires a separate search outside of the workflow). Additionally, POC CDST that are optimally integrated into the workflow and provide a time-saving benefit have been shown to improve physician practice patterns [29]. The utilization of a CDST to reduce inappropriate imaging referrals is not a new concept. In fact, a retrospective study of CT and MRI examinations from primary care physicians in 2010 found 26% of examinations to be inappropriate [30]. Blackmore and colleagues (2011) then demonstrated the use of CDS built into ordering systems reduced the utilization rate of MRI lumbar spine for low back pain, MRI head for headache, and CT sinus for sinusitis [18]. Challenges faced with available referral guidelines such as iGuide and iRefer include lack of awareness, access, and adherence [31]. A recent study in Italy investigating the appropriateness of imaging in patients with hepatocellular carcinoma and cholangiocarcinoma found that utilization of the ESR iGuide would have resulted in a 21.6% healthcare savings by preventing inappropriate imaging [10]. Another study that compared integrating CDS within an electronic referral (eReferral) process to the standard fax method in Canada found eReferrals to be 13 times more likely to be necessary than those made through fax [32]. Moreover, they found that ordering the most appropriate imaging test increased by 10% in 1 year following implementation, compared to a 7% decrease with fax [32]. Similarly, in this current study, referrers were 2.4 times more likely to refer patients for an MRI neck when using the POC CDST. This change in referral behavior further manifested as a reduction in effective dose, or 13 equivalent chest x-rays saved per patient. Importantly, in 2021, there were 133,382 people in Ireland waiting for a CT or MRI scan. To eliminate this wait time, 5.3% of the current 2.5 million imaging volume would need to be removed. When only a subset of referrers for HN imaging were given access, the POC CDST in this study eliminated 8.2% of the advanced imaging volume, suggesting that on a national scale this tool could reduce wait times in Ireland significantly.

There are limitations to the current study. First, the study occurred within a tertiary referral hospital that specializes in head and neck cancer. It would have been of interest to see how POC CDST impacts referrals in other departments such as thoracic or abdominal imaging. The main benefit of MRI over CT in the patient clinical scenarios assessed in this paper is the reduced radiation dose to the patient. This study was focussed on the imaging outcomes, but it would have been interesting to quantify how many referrers changed or cancelled their request within the application after being shown the POC CDST feedback. Instead, the reduction in inappropriate referrals was inferred through the average change in ordering behavior and reduction in advanced imaging volume. Furthermore, this study, as with all observational research, could be affected by confounding variables. Particularly, we recognize the challenge in controlling for confounders such as, but not limited to, the impact of the HSE cyber-attack, and the widespread effects of the COVID pandemic. Such confounding variables could diminish or exaggerate the effect of the POC CDST. Further studies with larger sample sizes will help mitigate these variables and further identify the true effect of a POC CDST. Future work investigating the effect POC CDST may have on time to diagnosis and cancer staging would further elucidate the impact on patient care. Finally, referrers using the POC versus web-based pathways were not entirely independent. For one, referrers worked at the same institution and therefore continued to communicate with one another. Additionally, to prevent disruption to the referring physician workflow, the subset of referrers using the POC CDST were given the option of using either referring pathway. Therefore, a learning effect may be present, whereby a single referrer may change their behavior within the application and apply this knowledge in the web-based pathway, such that the actual effect of implementing a POC CDST may in fact be greater than the effect observed in this study.

The use of medical imaging has exponentially increased over the last decade, and with it, an increase in inappropriate imaging referrals. In 2021, there were 226,166 patients waiting for a scan in Ireland [4]. By implementing a POC CDST that incorporates imaging referral guidelines into the referring physician’s workflow, it is possible to significantly reduce inappropriate imaging, advanced imaging volume, and effective patient radiation dose exposure in addition to reducing carbon emissions. Implementation on a national scale could markedly impact patient waiting lists in Ireland.

## Data Availability

The datasets used and/or analyzed during the current study are available from the corresponding author on reasonable request.

## Abbreviations

ACR: American College of Radiology
CDST: Clinical decision support tool
CO_2_e: Carbon dioxide equivalent
eReferral: Electronic referral
ESR: European Society of Radiology
HN: Head and neck
HNC: Head and neck cancer
HSE: Health Service Executive
OR: Odds ratio
POC: Point of care
RIS: Radiology Information System
